# The Adverse Effect of Anxiety on Dynamic Anticipation Performance

**DOI:** 10.3389/fpsyg.2022.823989

**Published:** 2022-03-03

**Authors:** Pengfei Ren, Tingwei Song, Lizhong Chi, Xiaoting Wang, Xiuying Miao

**Affiliations:** ^1^School of Psychology, Beijing Sport University, Beijing, China; ^2^School of Physical Education, Yan’an University, Yan’an, China

**Keywords:** processing efficiency, table tennis, perceptual-cognitive skill, sport expertise, attention control theory

## Abstract

Anticipation is a crucial perceptual-cognitive skill in fast-ball sports, and the effect of high anxiety on performance has attracted more attention from sports psychologists. Related studies mainly focus on the effect of anxiety on influencing processing efficiency and attentional control (top-down vs. bottom-up) during information processing in sport. Attentional Control Theory (ACT) has been supported by several studies. However, these studies have been criticized by the low ecological validity of task design, such as neglecting the dynamic process of anticipation, and inadequate performance analysis, such as analyzing response accuracy and time separately. Using temporal occlusion paradigm, we tested ACT in a dynamic anticipation process. Eighteen skilled and eighteen less-skilled table tennis players were required to anticipate the serves of opponents under dynamic task constraints (early vs. late occlusion) and anxiety conditions (high vs. low anxiety). High cognitive state anxiety decreased processing efficiency (response time/response accuracy) for both groups whereas performance effectiveness (response accuracy) did not differ. In addition, it negatively affected processing efficiency in early anticipation compared with late anticipation tasks, suggesting that high cognitive state anxiety may have a greater impact on top-down attentional control. Our findings provide support for ACT and show that anxiety impairs anticipation efficiency and performance, possibly due to an ineffectively attentional shift from external kinematic cues to internal long-term working memory. Findings also have implications for the adaptation of attentional strategies and anxiolytic training.

## Introduction

The influence of anxiety on performance has attracted more attention from sports psychologists. Recently, researchers have tested assumptions on Processing Efficiency Theory (PET, Eysenck and Calvo, 1992) and Attentional Control Theory (ACT, Eysenck et al., 2007) to explore the effects of anxiety on information processing in the competitive sport context.

Anxiety, as one kind of emotion, may arise in response to a competitive situation. Anxiety is a multidimensional concept in two ways. On the one hand, like other emotions, anxiety has both a trait component and a state component. According to Cox (2012), “state anxiety is an immediate emotional state that is characterized by apprehension, fear, tension, and an increase in physiological arousal” (p. 157). Conversely, trait anxiety is a predisposition to perceive certain environmental situations as threatening and to respond to these situations with increased state anxiety (Spielberger, 1971). State anxiety is a situation-specific affective response to the environment and is influenced by trait anxiety (Endler and Kocovski, 2001). An athlete with a high level of trait anxiety is likely to respond to a competitive situation with a high state of anxiety. That is, the effect of anxiety on performance is finally reflected through the influence of state anxiety. In addition, compared to trait anxiety, state anxiety is easily managed and controlled. Exploring the effect of state anxiety on performance can also inspire more evidence-based practice. Accordingly, the present study focuses on state anxiety.

On the other hand, anxiety has both cognitive and somatic components. Cognitive anxiety is the mental component of anxiety caused by things such as fear of negative social evaluation, fear of failure, and loss of self-esteem, while somatic anxiety is the physical component of anxiety and reflects the perception of physiological responses, such as increased heart rate, respiration, and muscular tension (Cox, 2012). These components have different temporal changes as a sport event approaches. Cognitive anxiety fluctuates throughout the contest as the probability of success or failure changes, while somatic anxiety dissipates rapidly after the start of the performance (for details, refer to 7.3, p. 164; Cox, 2012). This means that cognitive anxiety has a long-term effect during sport performance. Moreover, both PET and ACT emphasize the effect of cognitive anxiety on perceptual-cognitive performance in sport. Accordingly, the present study focuses on cognitive state anxiety.

According to PET, anxiety depletes limited working memory resources, which makes it insufficient for coincident tasks. Anxiety can also motivate individuals to recruit additional resources to maintain performance by improving motivation. Processing efficiency, i.e., the ratio of performance effectiveness (response accuracy) to mental effort, decreases as individuals improve mental effort to maintain performance under high-anxiety conditions. Studies have also shown that processing efficiency is more negatively impacted than performance effectiveness under high-anxiety conditions (e.g., Cocks et al., 2016; Vater et al., 2016).

Eysenck et al. (2007) developed ACT to further explain the cognitive mechanism of the adverse influence of anxiety on performance. ACT suggests that anxiety impairs the central executive system of working memory by reducing working memory resources available for coincident tasks. Attention as a cognitive processing refers to the mental activity of humans taking in external information or attending to internal resources. In addition, it plays an important role in the complex cognitive processing and behavior. ACT also proposes that anxiety impairs goal-oriented (top-down) attentional control by impacting inhibition and shifting the function of the central executive system. Inhibition and shifting dysfunction create the processing under the predominance of stimulus-driven (bottom-up) attentional control. This means that anxious individuals draw more attentional resources to the threat-related or salient stimulus (e.g., internal worrisome thoughts or external environment) and disturb themselves to concentrate on the goal or expectation formulated by prior experience and knowledge.

Based on PET and ACT, several studies have tested the effects of anxiety on sports performance. Cocks et al. (2016) examined the impact of anxiety on anticipation using a dynamic and time-constrained tennis task and found that processing efficiency significantly decreased under high compared to low-anxiety conditions. However, performance effectiveness did not differ. Vater et al. (2016) had a similar finding by using football decision-making tasks. They found that mental effort significantly increased under high-anxiety conditions. These findings suggest that anxiety elicits greater decrements in processing efficiency than performance effectiveness and provide solid support for PET.

To test the impact of anxiety on players’ attentional processes (high- and low-level cognitive processes), Cocks et al. (2016) elicited high- and low-level cognitive processes by manipulating the availability of postural, shot sequencing, and court position information in a tennis anticipation paradigm. The findings showed that high anxiety was most detrimental to processing contextual information only (top-down cognitive process) compared to performance in processing postural information only (bottom-up cognitive process), suggesting that anxiety may have a greater impact on top-down attentional control. Vater et al. (2016) investigated how anxiety influenced visual search strategies (attentional control) by manipulating far and near soccer decision situations. Their results showed that high anxiety influenced search strategies, with higher-skilled athletes showing a decrease in the number of fixations for far situations under high anxiety compared to low-anxiety conditions when compared with lower-skilled athletes. Both studies provide support for ACT with high anxiety impairing top-down attentional control.

The studies mentioned above provide partial support for PET and ACT. However, there are also significant shortcomings in sports performance analysis and representative task designs.

When rating processing efficiency, current researches generally use the ratio of performance effectiveness, such as anticipation or decision-making accuracy, to mental effort or only to the score of mental effort. However, there is a lack of performance analysis-combined accuracy with response time. In fact, a fast response is a basis for successful performance, especially in fast-ball sports. For example, with a tennis court length of 23.77 m, a tennis serve speed can reach 240 km/h (66.67 m/s) or higher (Gillet et al., 2009). Hence, receiving players need to complete the receiving motion in less than 0.357 s. Therefore, to examine the effect of anxiety on the anticipation of fast-ball receiving and serving, both anticipation accuracy and response time must be considered.

The study on speed-accuracy trade-off has proposed the indicator of inverse efficiency score (IES, the ratio of response time to accuracy; Bruyer and Brysbaert, 2011). The suggestion that higher IES indicates poorer processing efficiency or sports performance has received much attention and recognition from researchers on athletic anticipation (e.g., Abreu et al., 2012; Liu et al., 2017). Consequently, when examining processing efficiency, in addition to choosing mental effort, it is necessary to add IES to more comprehensively and effectively examine the effect of high anxiety on anticipation processing efficiency and evaluate the quality of anticipation performance.

Currently, researchers increasingly highlight the ecological validity of experimental task designs when conducting athletic anticipation studies, i.e., experimental tasks should adequately represent the real performance environments for better generalization and application of the findings (Runswick et al., 2018). For example, Cocks et al. (2016) classified the information of tennis landing anticipation into postural, shot sequencing, and court position information. They found that experimental tasks with only one kind or part of this information are different from the actual motor tasks where all information is combined. Drawing on a badminton serve anticipation task and an auditory selection response task (dual-task paradigm), Alder et al. (2018) explored the effects of high-anxiety on athletic anticipation, attentional resource allocation, and visual search strategy. However, the manipulation of these auditory tasks in laboratory remained significantly different from the actual performance scenarios. Runswick et al. (2018) examined the impact of anxiety on the processing of various information in sports anticipation by manipulating contextual information, such as the ball and the player’s field position using an *in situ* task, and the ecological validity of their study was improved to some extent. Currently, researches mostly discuss the effects of anxiety on perceptual-cognitive processing of different kinds of athletic information from a static perspective, ignoring the dynamic perceptual-cognitive process (for details, refer to Müller and Abernethy, 2012), such as anticipation in sports. That is, few studies pay attention to the effects of anxiety on the anticipation performance at different time points in the real scenarios of fast-ball sports.

Anticipating opponent’s movements and ball flight trajectories is particularly important for successful performance in fast-ball sports (Williams and Jackson, 2019). In an actual game, due to the high temporal and spatial pressure, the receiver is often unable to wait until the serving player fully completes a serving action to make a prediction, hence, he/she constantly anticipates the shot landing position during the unfolding events of a serve. Since early anticipation is based on incomplete and limited motor cues (Müller and Abernethy, 2012), athletes could draw on their prior motor experience to form expectations about the direction of a serve. A meta-analysis including 42 studies of perceptual-cognitive skill indicated that experts are better than non-experts in picking up perceptual cues, anticipating the landing area of the ball, and making an action decision, characterized by better response accuracy and faster response time (Mann et al., 2007). This means that expert athletes can consistently demonstrate superior perceptual-cognitive skills in athletic performance.

According to the long-term working memory theory (Ericsson and Kintsch, 1995; Williams and Ericsson, 2005), higher-skilled athletes have more motor experience and knowledge. They can turn attention to internal long-term working memory in the early stage of the opponent’s serve (incomplete motor cues). According to ACT, prior experience and knowledge help to make a goal-oriented and top-down attentional control. As a serve event unfolds, the motor cues provide increasingly complete and definitive information. Hence, athletes need to shift attention from internal long-term working memory to external action cues, i.e., to form a stimulus-driven and bottom-up attentional control. As these attentional control skills are expertise-dependent, it seems significant to test whether the effects of anxiety interact with skill level.

To summarize, PET suggests that cognitive state anxiety (e.g., worrisome thoughts) occupies and negatively affects working memory, while ACT further investigates how cognitive state anxiety affects working memory. ACT suggests that cognitive state anxiety impairs the central executive system of working memory, especially inhibition and shifting function.

Based on ACT, long-term working memory theory, and the important role of anticipation in fast-ball sports, this study will examine the differences in anticipation performance between skilled and less-skilled athletes under high- and low-anxiety conditions in early and late anticipation tasks, and whether skill level can moderate the relationship between anxiety and anticipation performance. We propose two following hypotheses: (1) With the increase of anxiety level, processing efficiency in anticipation tasks decrease significantly, while performance effectiveness remain; and (2) with the increase of anxiety level, the processing efficiency and anticipation performance of athletes in early anticipation, especially in skilled athletes, will be disrupted to a greater extent compared to in late anticipation.

In the current study, we make a novel attempt to examine the effects of anxiety on attentional control in anticipation by using table tennis serving tasks at different occlusion times. As a fast-ball sport, table tennis provides a good experimental context, requiring athletes to have the ability to anticipate quickly, accurately, and stably. The role of receiving a serve for the technical and tactical initiative is getting more and more attention from athletes and coaches (Cheng and Yang, 2014; Zhou and Zhang, 2019). In addition, ACT has not found support in table tennis anticipation tasks. Exploring the effect of high anxiety on receiving and serving in fast-ball sports could broaden and deepen the understanding of the relationship between the psychological state of athletes and athletic performance, and provide scientific evidence for guiding table tennis training and competition. In addition, exploring the effects of high anxiety on performance by using table tennis anticipation tasks at different occlusion times could test ACT in a more ecological way and provide more empirical evidence.

## Materials and Methods

### Participants

A prior analysis was used and the sample size was calculated by G*Power 3.1 (Faul et al., 2009). Based on the medium effect size (*f* = 0.25), given an α of 0.05, and a statistical power (1-β) of 0.95, 36 players took part in this study. Eighteen college table tennis players (10 males and 8 females; *M*_age_ = 22.8 years, *SD* = 2.58; *M*_experience_ = 13.1 years, *SD* = 5.11) were recruited for the higher-skilled group, with their highest playing level ranging from regional (*n* = 7) or provincial (*n* = 8) to national (*n* = 3) level and 9.83 training hours per week (*SD* = 3.67). The lower-skilled group consisted of 18 college students who majored in physical education of table tennis (all male; *M*_age_ = 20.5 years, *SD* = 1.43; *M*_experience_ = 2.1 years, *SD* = 0.78). They learned to play table tennis for less than 2 years, with 5.4 training hours per week (*SD* = 2.14), and only participated in campus table tennis games. All participants were right-handed and reported to be in good health, had no mental illness, and had normal or corrected to normal vision. Participants were tested individually. Before testing, all participants provided written informed consent. The study was approved by the university ethics committee.

### Design

The study employed a 2 (skill level: skilled/less-skilled) × 2 (anxiety: high/low) × 2 (occlusion time: early/late) mixed-factor design. Anxiety and occlusion time were within-participants factors. Performance effectiveness (response accuracy), processing efficiency (IES: response time/accuracy; and mental effort ratings), and response time were the dependent variables.

### Materials

#### Trait Anxiety

State anxiety is influenced by trait anxiety (Endler and Kocovski, 2001). Previous researchers proposed that individual differences of trait anxiety may cause the differences of state anxiety induced by the same anxiety-inducing procedure (for details, refer to the Discussion, Vater et al., 2016). Moreover, trait anxiety may interfere with the executive function of participants, such as inhibition function (Sun and Zhang, 2015) and shifting function (Wu et al., 2021). To test the effectiveness of the anxiety induction procedure and reduce the adverse effects of individual differences, trait anxiety was set as the control variable.

Trait anxiety was measured using the Trait Anxiety Inventory (TAI) from the State-Trait Anxiety Inventory (Chinese translation edition, Spielberger et al., 1983; Wang et al., 1999). TAI has 20 items, and each requires a response on a 4-point scale from “Almost Never” to “Almost Always.” The scores of TAI range from 20 to 80, and higher scores indicate greater trait anxiety. The coefficient of internal consistency reliability (Cronbach’s α) in the study is 0.73.

#### Competitive State Anxiety

Martens et al. (1990) developed competitive state anxiety as multidimensional anxiety theory that included cognitive, somatic anxiety, and self-confidence. Referring to related studies (e.g., Runswick et al., 2018; Broadbent et al., 2019), we used the Mental Readiness Form-Likert (MRF-L; Krane, 1994) to measure competitive state anxiety. MRF-L has three items to assess cognitive anxiety [from “not worried” (1) to “worried” (11)], somatic anxiety [from “not tense” (1) to “tense” (11)], and self-confidence [from “not confident” (1) to “confident” (11)], respectively, with participants responding on an 11-point Likert scale.

#### Mental Effort

The Rating Scale for Mental Effort (RSMF; Zijlstra, 1993) was used to assess mental effort. RSMF is a sensitive indicator to assess mental effort (Broadbent et al., 2019). It requires participants to provide a number from “0” to “150” to denote their perceived mental effort, with “0” corresponding to not at all effortful, “75” corresponding to moderately effortful, and “150” to very effortful.

#### Recording, Editing, and Screening of Anticipation Tasks

**FIGURE 1:**
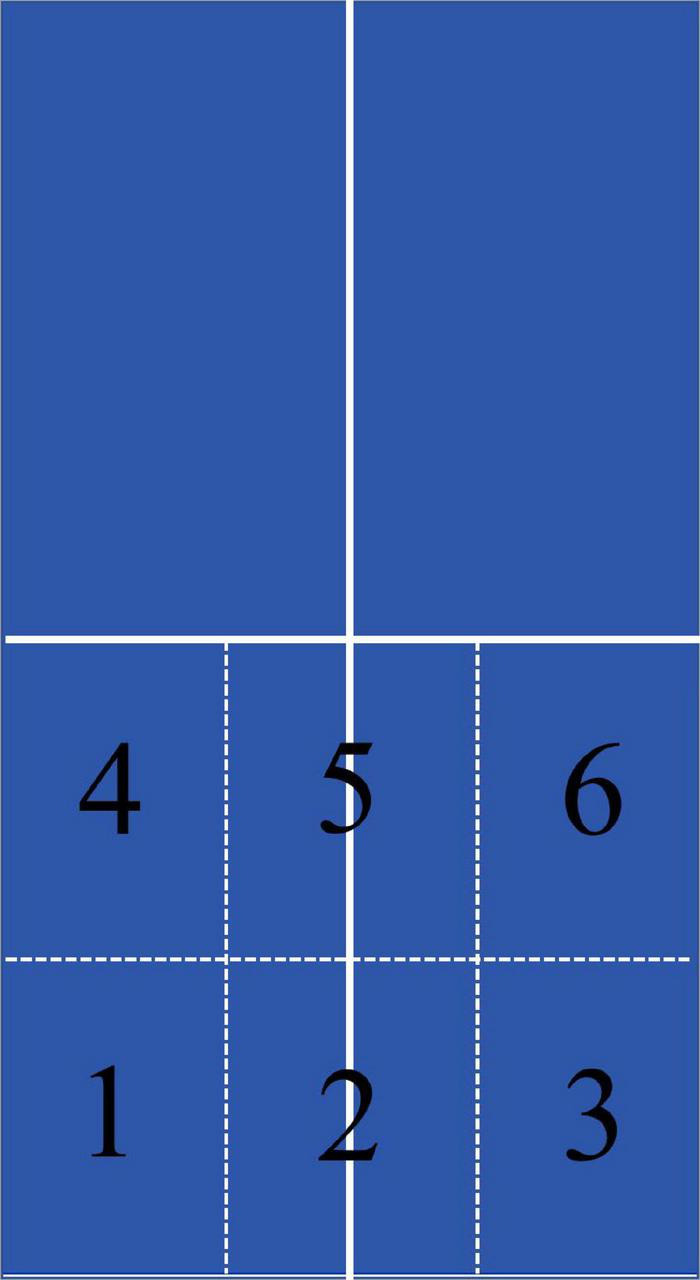
Landing target area division.

Four provincial-level table tennis players (4 males; 2 right-handed; *M*_age_ = 20.3 years, *SD* = 2.52; *M*_experience_ = 12.8 years, *SD* = 3.23) were recruited to record table tennis serves. In accordance to the rules of the International Table Tennis Federation, they used an uncovered serve in the side position. They were required to use forehand backspin or side backspin technique just like in real competition, with six landing zones (left, middle, and right area near and far from the net, as shown in Figure 1). Under the requirements of keeping the speed, strength, and flight arc of the serves as stable as possible, each of them completed 10 serves at each landing zone, and a total of 240 serve videos were recorded. To simulate the perspective of real competition, the camera support was placed on the extension line of the centerline on the table, two meters away from the table, and about 4.75 m away from the server. The camera lens was 1.7 m high from the ground. The resolution of the videos was 1,920 × 1,080, with a frame rate of 30 fps.

Four table tennis graduate students (two males and right-handed; provincial-level; *M*_age_ = 28.2 years, *SD* = 3.32; *M*_experience_ = 10.8 years, *SD* = 2.51) were recruited to screen videos. The quality of the serve was evaluated by four aspects on a 5-point scale, i.e., the server’s position, ball throwing, racket leading, and hitting. Finally, the videos with the quality score of each landing zone in the top 1/2 were selected (the average score of each zone is above 4.15), that is, each player had 5 videos at each landing zone, and a total of 120 videos were screened out.

The videos were edited by the software Kinovea 0.9.1 (a free video player for sports analysis created and developed by Joan, 2018) without any sound. An anticipation video clip started at the moment of the ball throw during the serve period, where difficult tasks (early occlusion) ended at the moment when the ping-pong ball touched the home side of the table (lasting about 1,230 ms) and easy tasks (late occlusion) ended at the moment when the ping-pong ball bounced right above the net (lasting about 1,570 ms). Each serve video was edited into an easy task and a difficult one, and a total of 240 videos were generated. Considering the factors of server actors and their handedness, these selected videos evenly distributed in four experimental conditions, namely, early and late occlusion in high-anxiety condition and early and late occlusion in low-anxiety condition. Ultimately, there were 60 videos in each condition.

### Apparatus

A Nikon D7100 camera was used for recording. An HP laptop, which was configured with a 15.6-inch screen (resolution of 1,920 × 1,080), 16 g memory, and Intel I5-7300HQ CPU (frequency of 3.25 GHz), was used to edit videos, execute the experimental program, collect data, and do statistical analysis. The experimental data input device was a numeric keypad that only contained numbers 1–6. Four serve actors were provided with uniform sports T-shirts.

E-prime 2.0 (Psychology Software Tools, Inc. [E-Prime 2.0], 2015 Pittsburgh, PA) was used to present the stimuli and record the participants’ responses. In addition, a whiteboard (70 cm × 50 cm) was used to record participants’ scores in the anticipation tasks and a picture (A4 size) showed a description of the monetary reward.

Using an EPSON projector (CH-TW610) with 1080P resolution and a brightness of 3,000 lumens, video clips were projected onto a 100-in screen, creating an image size of 1.975 m width × 1.48 m height. The lower edge of the screen was 0.85 m from the ground. Participants were asked to stand 1.1 m away from the screen, such that the participant could subtend a similar visual angel as a real game.

### Anxiety Induction

Referring to related studies (e.g., Cocks et al., 2016; Vater et al., 2016; Alder et al., 2018; Runswick et al., 2018; Broadbent et al., 2019), we jointly used camera shooting, error feedback, real-time feedback, score ranking, and monetary rewards to induce competitive state anxiety. Under high-state anxiety conditions, the camera recorded the entire experimental process. Participants were informed that their anticipation performance would be shown in the classroom as an instructional video (not actually shown in any occasion). Meanwhile, participants were given five random error feedbacks out of six practice trials. Just when they finished all the tasks in high-anxiety conditions, participants were given their anticipation scores by being shown on the whiteboard and were informed that experimental rewards would be given based on their score ranking.

### Response Accuracy and Response Time

After each video was occluded, participants were requested to indicate where on the table they thought the ping-pong ball would land by pressing 1-6 on the keyboard (the arrangement of the number keyboard is the same as shown in Figure 1. **Response accuracy** was measured as the percentage of correct responses produced by the participants.

**FIGURE 2:**
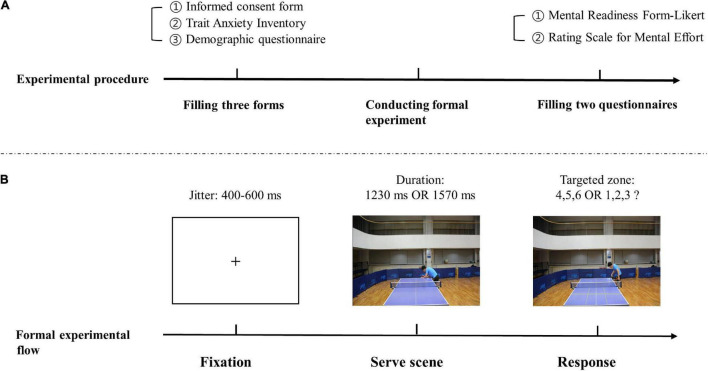
Experimental procedure and formal experimental flow. **(A)** Shows the experimental procedure. Participants first completed three forms before the experimenter explained the experimental procedure to them. Then, participants conducted the formal experiment in low or high-anxiety conditions in one experimental time. Lastly, they filled two questionnaires. **(B)** Shows a formal experimental flow. After low or high anxiety induction procedure, the experiment started from a “+” appearing on the screen as a reminder before the anticipation video played. After the video occlusion, three yellow lines would appear on the last frame of the video to show six targeted zone. Then, participants were asked to press the appropriate key.

**Response time** was defined as the time (ms) between the point of video occlusion (the last frame of a video with yellow lines appears as shown in Figure 2) and the onset of pressing the number keyboard.

### Procedure

The experiment was conducted in a table tennis court. When the participants arrived at the court, they were required to complete an informed consent form, a demographic questionnaire, and a trait anxiety inventory. Then, half of them participate in the low-anxiety conditions before completing the high-anxiety experiment at an interval of 2–3 days. The other half experimented in the opposite order and at the same interval. The order of high- and low-anxiety conditions conducted for participants was at random, according to a random number table with only 0 and 1.

After, the experimental procedure was explained to the participants. They first completed six practice trials and received feedback on correctness and response time. The half of the 240 test stimuli were presented in 12 blocks of 10 trials under the high- or low-anxiety conditions. Participants were given a 10-s break between each block and a 2-min break for every 6 blocks. There were 4 task conditions (right- and left-handed serves in early and late occlusion) in each anxiety condition. The presentation order of early and late occlusion tasks was random among participants, and test stimuli of two handedness conditions in one occlusion condition were also randomly presented. Each anxiety condition took no longer than 30 min to complete. The experimental procedure and formal experimental flow are shown in Figure 2.

### Data Analysis

Statistical analyses were conducted with (JASP 0.14, Amsterdam, The Netherlands) (Love et al., 2019). A general linear model is used to analyze the data. Before conducting main analyses, an independent sample *t*-test was undertaken to eliminate trait anxiety difference between two groups. In addition, four 2 × 2 repeated measures ANOVA were performed to examine the effects of anxiety induction, with anxiety conditions and skill level as independent variables and cognitive anxiety, somatic anxiety, self-confidence, and mental effort as dependent variables, respectively. Main analyses consisted of three separate 2 × 2 × 2 [anxiety (high/low), skill level (skilled/less-skilled) and occlusion time (early/late)] repeated measures of ANOVA with response accuracy, response time, and inverse efficiency score as dependent variables. Significant interactions were calculated using *post hoc* tests with Bonferroni corrections to avoid the inflation of Type 1 error. Effect sizes were evaluated using partial eta squared values (η*_*p*_*^2^) and Cohen’s *d*. The significant level (α) was set at 0.05.

## Results

### Anxiety Manipulation Check

#### Trait Anxiety

Independent sample *t*-test revealed a non-significant effect of trait anxiety, with the skilled group reporting no significantly different ratings (41.8 ± 6.17; i.e., *M* ± *SD*, the same hereinafter) in comparison to the less-skilled group [(38.6 ± 5.84), *t*(34) = 1.64, *p* = 0.11].

#### Competitive State Anxiety and Mental Effort

The descriptive statistics of scores on each subscale of MRF-L and RSMF for both groups in high- and low-anxiety conditions are shown in Table 1.

**TABLE 1:** Mean (SD) scores on each Mental Readiness Form-Likert (MRF-L) subscale and Rating Scale for Mental Effort (RSMF) for both groups in high- and low-anxiety conditions.

Anxiety	Skill level	Cognitive anxiety	Somatic anxiety	Self-confidence	Mental effort
High	Skilled	5.2 (2.66)	4.8 (2.56)	7.0 (2.66)	89.4 (26.78)
	Less-skilled	5.9 (2.37)	5.3 (1.94)	6.7 (2.00)	93.9 (15.96)
Low	Skilled	3.3 (2.11)	3.3 (2.32)	8.1 (2.21)	75.6 (26.62)
	Less-skilled	4.5 (2.18)	4.3 (2.49)	7.4 (2.06)	84.7 (17.86)

Analysis of variances (ANOVAs) revealed significant main effects of anxiety conditions for all the dependent variables. Participants reported significantly higher cognitive anxiety (5.5 ± 2.51) during high-anxiety conditions in comparison to low anxiety [(3.9 ± 2.20), *F*_(1,34)_ = 21.62, *p* < 0.001, η*_*p*_*^2^ = 0.39]. Similarly, participants reported significantly higher somatic anxiety [(5.1 ± 2.25) vs. (3.8 ± 2.43), *F* (1,34) = 10.12, *p* = 0.003, η*_*p*_*^2^ = 0.23], greater mental effort [(91. 7 ± 21.84) vs. (80.1 ± 22.82), *F* (1,34) = 26.7, *p* < 0.001, η*_*p*_*^2^ = 0.44], and less confidence [(6.8 ± 2.32) vs. (7.7 ± 2.13), *F* (1,34) = 7.52, *p* = 0.010, η*_*p*_*^2^ = 0.18] in high-anxiety conditions compared to low-anxiety conditions. The main effect of skill level and the interaction effect between anxiety and skill level were non- significant.

### Response Accuracy

There was a significant main effect of occlusion time [*F*_(1,34)_ = 94.1, *p* < 0.001, η*_*p*_*^2^ = 0.74]. Participants recorded higher accuracy scores in the late occlusion tasks (0.69 ± 0.11) than in the early occlusion tasks (0.54 ± 0.14). Also, a main effect of skill level was observed [*F*_(1,34)_ = 14.8, *p* < 0.001, η*_*p*_*^2^ = 0.30]. The skilled group (0.67 ± 0.14) was significantly more accurate than the less-skilled (0.56 ± 0.13). Moreover, a significant anxiety condition × occlusion time interaction was found [*F*_(1,34)_ = 5.9, *p* = 0.021, η*_*p*_*^2^ = 0.15]. All other effects were non-significant, as shown in Table 2.

**TABLE 2:** ANOVA results for response accuracy.

Statistic Effects	*F*	*df*	*p*	η*_*p*_*^2^
Anxiety	<0.01	(1,34)	0.940	<0.01
Skill level	14.8	(1,34)	< 0.001***	0.30
Occlusion time	94.1	(1,34)	< 0.001***	0.74
Anxiety × occlusion time	5.9	(1,34)	0.021*	0.15
Anxiety × skill level	0.39	(1,34)	0.538	0.01
Skill level × occlusion time	0.53	(1,34)	0.471	0.01
Anxiety × occlusion time × skill level	0.09	(1,34)	0.766	<0.01

*p < 0.05, ***p < 0.001. η_p_^2^ = 0.01, small effect size; η_p_^2^ = 0.06, medium effect size; η_p_^2^ = 0.14, large effect size.

**FIGURE 3:**
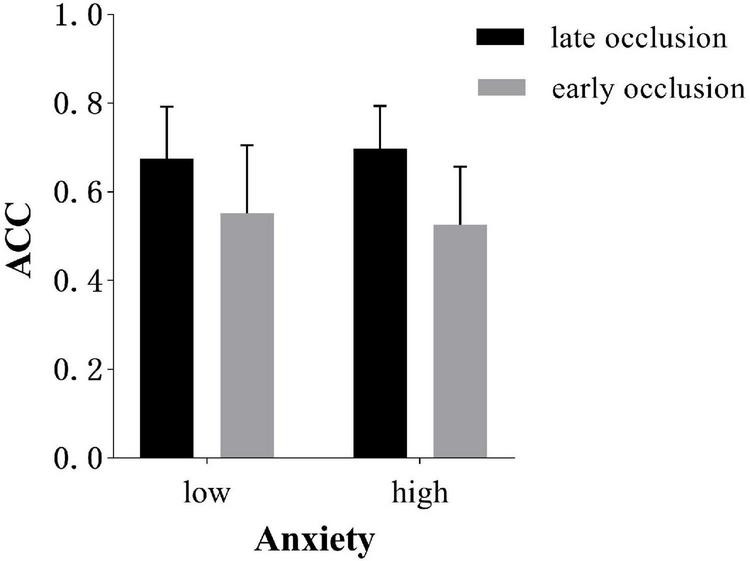
Response accuracy (*M* ± *SD*) of different occlusion time in low and high-anxiety conditions. Error bars are *SD*s.

The two-way interaction shows that there was a downward trend for anticipation accuracy [(0.55 ± 0.15) vs. (0.53 ± 0.13)]. with increasing levels of state anxiety in early occlusion tasks. On the contrary, in late occlusion tasks, there was an upward trend in low anxiety (0.68 ± 0.12) compared to high-anxiety conditions (0.70 ± 0.10). The two comparisons were non-significant, however, as shown in Figure 3.

### Response Time

There was a significant effect of anxiety [*F*_(1,34)_ = 21.7, *p* < 0.001, η*_*p*_*^2^ = 0.39]. Participants responded faster under low anxiety (804.5 ms ± 351.80) compared with high-anxiety conditions (950.1 ms ± 355.47). A significant main effect of occlusion time was observed, with participants responding faster in late (736.4 ms ± 284.52) than in early occlusion tasks [(1018.2 ms ± 373.68), *F*_(1,34)_ = 118.0, *p* < 0.001, η*_*p*_*^2^ = 0.78]. Also, there was a significant main effect of skill level [*F*_(1,34)_ = 7.5, *p* = 0.010, η*_*p*_*^2^ = 0.18] as shown in Table 3. The skilled group (754.7 ms ± 321.35) responded faster than the less-skilled (999.9 ms ± 356.53).

**TABLE 3:** ANOVA results for response time.

Statistic effects	*F*	*df*	*p*	η_p_^2^
Anxiety	21.7	(1,34)	< 0.001***	0.39
Skill level	7.5	(1,34)	0.010*	0.18
Occlusion time	118.0	(1,34)	< 0.001***	0.78
Anxiety × occlusion time	2.9	(1,34)	0.099	0.08
Anxiety × skill level	8.1	(1,34)	0.008**	0.19
Skill level × occlusion time	5.1	(1,34)	0.031*	0.13
Anxiety × occlusion time × skill level	0.1	(1,34)	0.725	< 0.01

*p < 0.05, **p < 0.01, ***p < 0.001. η_p_^2^ = 0.01, small effect size; η_p_^2^ = 0.06, medium effect size; η_p_^2^ = 0.14, large effect size.

In addition, two significant interactions of anxiety × skill level [*F*_(1,34)_ = 8.1, *p* = 0.008, η*_*p*_*^2^ = 0.19] and occlusion time × skill level [*F*_(1,34)_ = 5.1, *p* = 0.031, η*_*p*_*^2^ = 0.13] were observed. All other effects were non-significant.

**FIGURE 4:**
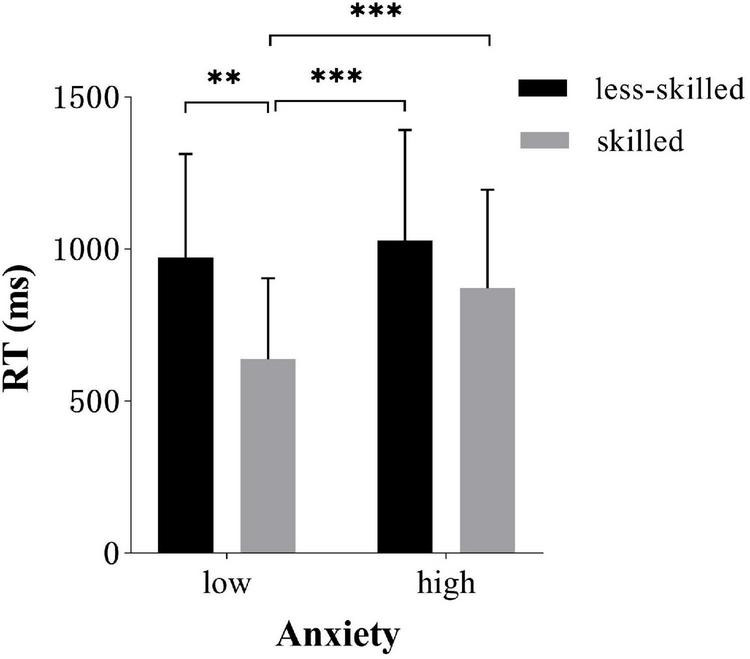
Response time (*M* ± *SD*) for two groups in low and high-anxiety conditions. Error bars are *SD*s. ^**^p < 0.01, ^***^p < 0.001.

*Post hoc* analysis of anxiety × skill level interaction revealed that the skilled responded faster in low anxiety (637.6 ms ± 267.26) than high-anxiety conditions [(871.8 ms ± 323.73), *t* = 5.3, *p* < 0.001, *d* = 0.79]. In contrast, the less-skilled did not show significant differences in response time under high anxiety (1028.3 ms ± 363.55) compared to low-anxiety (971.5 ms ± 341.94), as shown in Figure 4.

**FIGURE 5:**
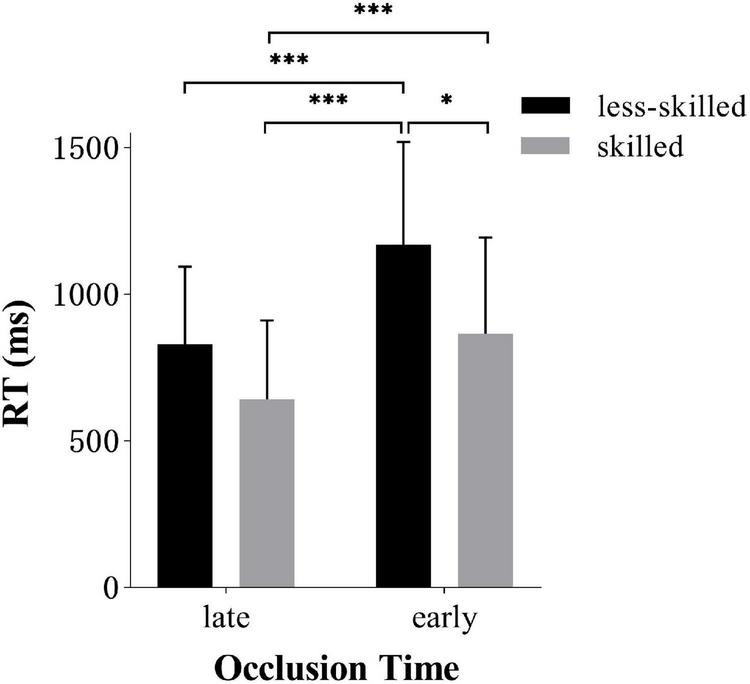
Response time (*M* ± *SD*) for two groups in early and late anticipation. Error bars are *SD*s. *p < 0.05, ^***^p < 0.001.

*Post hoc* analysis of occlusion time × skill level interaction revealed that the skilled (866.4 ms ± 327.24) responded faster in early occlusion tasks than the less-skilled [(1170.0 ms ± 349.58), *t*(34) = 6.1, *p* < 0.001, *d* = *0.90*]. However, the skilled did not show significant differences in late occlusion (643.0 ms ± 267.66) compared to the less-skilled group (829.7 ms ± 265. 68), as shown in Figure 5.

### Inverse Efficiency Score

There was a significant main effect of anxiety [*F*_(1,34)_ = 6.6, *p* = 0.015, η*_*p*_*^2^ = 0.16]. Participants were more efficient in low anxiety (1469.3 ± 986.68) compared with high-anxiety conditions (1,705.9 ± 959.89). A significant main effect of occlusion time was observed, with participants more efficient in late (1120.7 ± 578.12) than early occlusion tasks [(2054.5 ± 1070.95), *F*_(1,34)_ = 74.6, *p* < 0.001, η*_*p*_*^2^ = 0.69]. Also, there was a significant main effect of skill level [*F*_(1,34)_ = 16.9, *p* < 0.001, η*_*p*_*^2^ = 0.33], as shown in Table 4. The skilled group (1193.8 ± 621.87) was more efficient than the less-skilled (1981.36 ± 1105.50).

**TABLE 4:** ANOVA results for inverse efficiency score.

Statistic Effects	*F*	*df*	*p*	η*_*p*_*^2^
Anxiety	6.6	(1,34)	0.015*	0.16
Skill level	16.9	(1,34)	< 0.001***	0.33
Occlusion time	74.6	(1,34)	< 0.001***	0.69
Anxiety × occlusion time	3.83	(1,34)	0.058	0.10
Anxiety × skill level	3.93	(1,34)	0.055	0.10
Skill level × occlusion time	7.8	(1,34)	0.009**	0.19
Anxiety × occlusion time × skill level	<0.01	(1,34)	0.965	<0.001

*p < 0.05, **p < 0.01, ***p < 0.001. η_p_^2^ = 0.01, small effect size; η_p_^2^ = 0.06, medium effect size; η_p_^2^ = 0.14, large effect size.

Moreover, there was a significant interaction of occlusion time × skill level [*F*_(1,34)_ = 7.8, *p* = 0.009, η*_*p*_*^2^ = 0.19]. Also, two marginally significant interactions of anxiety × skill level [*F*_(1,34)_ = 3.93, *p* = 0.055, η*_*p*_*^2^ = 0.10] and anxiety × occlusion time [*F*_(1,34)_ = 3.83, *p* = 0.058, η*_*p*_*^2^ = 0.10] were observed. All other effects were non-significant.

**FIGURE 6:**
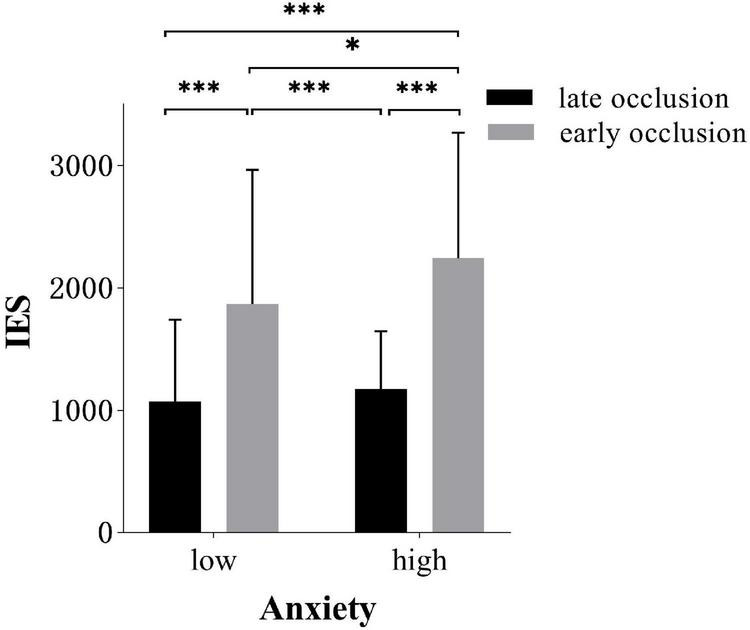
Inverse Efficiency Score (IES; *M* ± *SD*) of different occlusion time in low and high-anxiety conditions. Error bars are *SD*s. *p < 0.05, ^***^p < 0.001.

*Post hoc* test of anxiety × skill level interaction revealed that the skilled responded more efficiently in low anxiety (984.0 ± 460.94) than high-anxiety conditions [(1403.7 ± 679.82), *t*(34) = 3.2, *p* = 0.017, *d* = 0.72]. However, there was no difference for the less-skilled group between two anxiety conditions [(2008.1 ± 1082.77) in high anxiety (1954.63 ± 1,111.97) in low anxiety], as shown in Figure 6.

**FIGURE 7:**
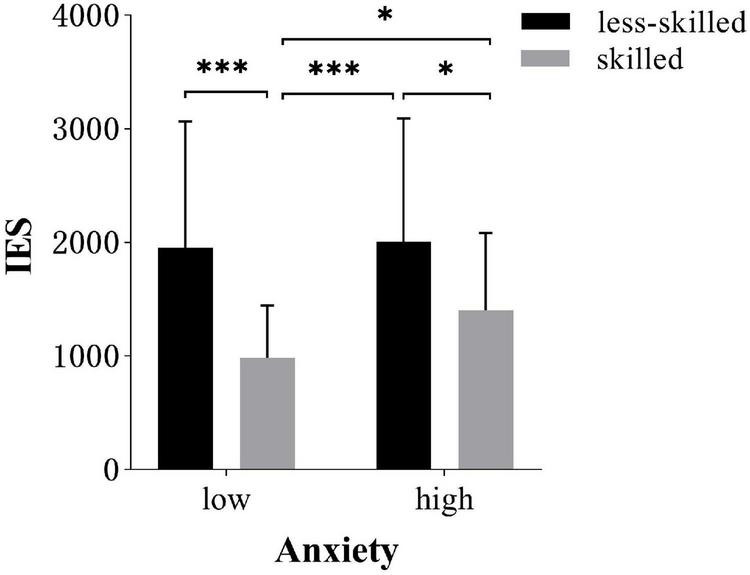
IES (*M* ± *SD*) for two groups in low and high-anxiety conditions. Error bars are *SD*s. *p < 0.05, ^***^p < 0.001.

*Post hoc* test of anxiety × occlusion time interaction showed that participants responded more efficiently with low anxiety (1867.4 ± 1,096.98) than high-anxiety conditions (2241.6 ± 1025.31) in early occlusion tasks [*t*(34) = 3.2, *p* < 0.012, *d* = 0.35]. However, there was no difference for participants in late occlusion between two anxiety conditions [(1170.2 ± 476.91) in high anxiety (1071.2 ± 667.47) in low anxiety], as shown in Figure 7.

**FIGURE 8:**
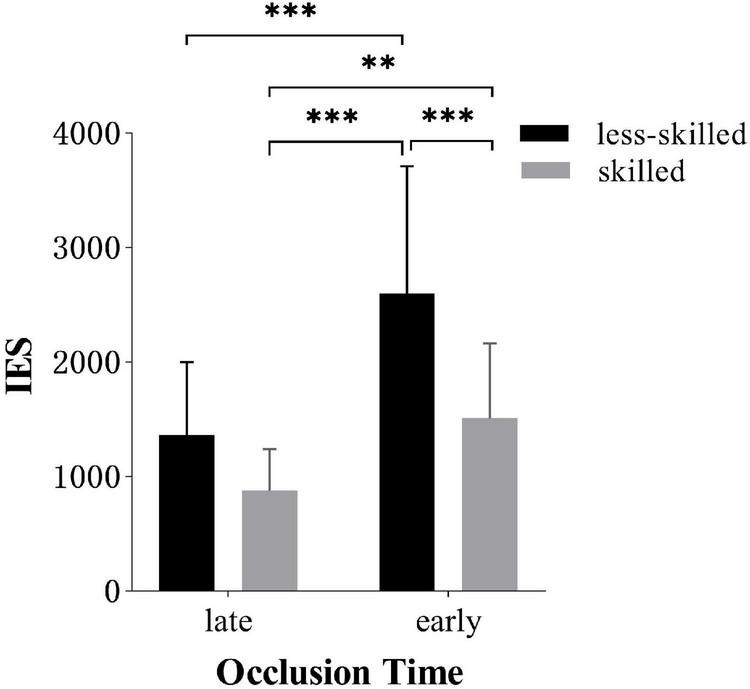
IES (*M* ± *SD*) for two groups in early and late anticipation. Error bars are *SD*s. ^**^p < 0.01, ^***^p < 0.001.

*Post hoc* analysis of skill level × occlusion time interaction showed that the skilled (1,510.0 ± 655.95) responded more efficiently than the less-skilled (2599.1 ± 1112.96) in early occlusion tasks [*t*(34) = 5.0, *p* < 0.001, *d* = 1.19]. However, there was no significant difference for participants in late occlusion [(877.7 ± 364.08) for the skilled (1363.7 ± 639.16) for the less-skilled], as shown in Figure 8.

## Discussion

Attentional control theory was retested using early and late occlusion anticipation tasks of table tennis. As anxiety levels increased, we predicted a more significant decrease in processing efficiency of players’ anticipation performance compared to performance effectiveness. In addition, anticipation performance was more disrupted among players in early anticipation tasks, especially in skilled players, compared to late anticipation.

Using a 2 (skill level: skilled/less-skilled) × 2 (anxiety: high/low) × 2 (Occlusion time: early/late) mixed-factor design, we examined the effects of competitive state anxiety on processing efficiency (IES, ratio of reaction time to accuracy) and performance effectiveness (response accuracy) of anticipating landing location of table tennis serves. Results show that high cognitive state anxiety reduces the processing efficiency of players in anticipation tasks, but does not disrupt anticipation accuracy. In addition, high cognitive state anxiety disrupts early anticipation performance for both skilled and less-skilled players. By creating a competitive environment and evaluation threat and adding error feedback and money reward, high cognitive state anxiety was successfully induced in athletes after excluding differences of trait anxiety between skilled and less-skilled players. The cognitive anxiety of participants was more pronounced with a large effect size, consistent with PET, which suggests that high anxiety triggers worried thoughts in individuals and increases working memory load (Eysenck and Calvo, 1992). In addition, an anxiety-inducing procedure causes elevated somatic anxiety and a decrease in self-confidence. The effects of induced anxiety in the present studies were better than those of related studies (e.g., Cocks et al., 2016; Vater et al., 2016; Alder et al., 2018; Runswick et al., 2018; Broadbent et al., 2019) in terms of the effect size, most likely due to the combined use of anxiety-inducing methods from the studies mentioned above that induced higher levels of state anxiety.

High cognitive state anxiety reduced the processing efficiency of players in table tennis anticipation tasks but did not reduce their response accuracy. Specifically, the mental effort of athletes in high-anxiety conditions was significantly higher and had large effect size. At the same time, high anxiety did not lead to a significant decrease in anticipation accuracy, i.e., athletes’ processing efficiency was significantly affected by high cognitive state anxiety, but performance effectiveness was not significantly affected. Hence, hypothesis (1) was supported. This is consistent with results generally confirmed by related studies (e.g., Cocks et al., 2016; Vater et al., 2016; Broadbent et al., 2019) and can be explained by PET and ACT.

Processing Efficiency Theory (PET) and ACT (Eysenck and Calvo, 1992; Eysenck et al., 2007) suggest that anxiety triggers worried thoughts in individuals and occupies working memory resources, resulting in insufficient working memory resources for the current task. On the other hand, anxiety also improves motivation and drives individuals to exert more effort, i.e., by recruiting more cognitive resources devoted to the current task to maintain performance. As a result, individuals in high anxiety would reduce their processing efficiency.

High cognitive state anxiety reduced the processing efficiency of table tennis players in anticipation tasks, which was also supported by the prolonged response time. Players in high anxiety responded more slowly than those in low anxiety, and the effect size was large. It is speculated that with the improvement of state anxiety, worried thoughts induced by cognitive anxiety occupy some working memory resources. In addition, by prolonging response time to increase the investment of cognitive resources, there was no significant decrease in anticipation accuracy at the cost of reduced processing efficiency in anticipation tasks.

High cognitive state anxiety reduced athletes’ performance in early anticipation and was not moderated by skill level. This finding was a moderate effect size and partially supported the hypothesis (2). Athletes in high anxiety had significantly higher inverse efficiency scores in early anticipation than those in low anxiety. High anxiety significantly reduced athletes’ performance in early anticipation tasks, while performance in late anticipation was not affected. ACT suggests that high anxiety drives individuals to attend to threat-related stimuli (Eysenck et al., 2007), but the absence of overtly threatening stimuli in the anticipation tasks precludes the influence of threat-related stimuli. The theory also suggests that high anxiety causes individuals to have worried thoughts (Eysenck and Calvo, 1992; Eysenck et al., 2007), which can be verified by the successful induction of high cognitive anxiety. These worrying thoughts occupy the limited working memory resources available for the current anticipation tasks, and the occupation of working memory resources in turn disrupts participants’ ability to inhibit irrelevant stimuli (Eysenck et al., 2007). Researchers found that the attentional strategies of athletes were changed in high anxiety compared to low-anxiety conditions. Compared with low-anxiety conditions, athletes employ a strategy of more fixations and shorter fixation duration in high anxiety (Wilson et al., 2009; Runswick et al., 2018), which probably means that athletes attend to more irrelevant stimuli.

On the other hand, the occupation of working memory resources caused by high anxiety also prevented participants from flexibly shifting attention between different stimuli (Eysenck et al., 2007). According to the long-term working memory theory (Williams and Ericsson, 2005) and the conceptual model of motor anticipation (Müller and Abernethy, 2012), the internal and external stimuli in the anticipation process mainly involve the athletes’ internal long-term working memory and external action and ball flight cues. That is, high anxiety destroys the inhibition of worried thoughts and shifts functions between internal working memory and external cues.

In contrast to late occlusion tasks, action and ball flight cues are more incomplete in early anticipation, and athletes are more likely to constantly shift attention between expectations of where the ball will land based on their motor experience and external environment (actual action and ball flight trajectory). This view is consistent with recent researchers’ argument that the motor anticipation process is consistent with Bayesian theory, which suggests that athletes could combine ongoing expectations and dynamic environmental information to make a decision in sport under time pressure (Yarrow et al., 2009; Gredin et al., 2018). Therefore, we speculate that high anxiety disrupts the reasonable and efficient allocation of attentional resources between internal and external stimuli for athletes. Thus, performance in early anticipation, which is demanding in attentional resource allocation, is adversely affected by high anxiety. It is speculated that high anxiety disrupts the goal-directed, top-down attentional control of athletes in anticipation tasks.

In addition, the effect of sports expertise has also been verified. Compared with less-skilled players, skilled players have better anticipation performance both in high and low anxiety. Skilled players have higher anticipation accuracy (large effect size), shorter response time (large effect size), and lower inverse efficiency score (large effect size), indicating that the skilled can make better use of action posture and ball flight cues in anticipation. These results can be explained by more refined perceptual-cognitive skills of skilled players (Mann et al., 2007; Williams and Jackson, 2019). Experts have domain-specific knowledge structures that result in tasks being completed with fewer demands on working memory (Ericsson and Kintsch, 1995). These lower demands on working memory may allow the skilled to redistribute attentional resources to internal and external stimuli under high anxiety. In contrast, the unskilled players with high demands on working memory are not likely to redistribute attentional resources under high-anxiety conditions.

This study also found the interaction between skill level and state anxiety. High anxiety slowed down the response of skilled players and reduced their processing efficiency. However, the effect was not found in less-skilled players. According to the oral report of athletes, researchers found that the skilled players use more cognitive expressions than the less-skilled players in sports anticipation (Roca et al., 2013). These cognitive expressions based on memory representation help to guide athletes’ visual attention. Moreover, systematic differences in visual search behaviors were also observed, with experts using fewer fixations of longer duration, including prolonged quiet eye periods, compared with non-experts or the less-skilled players (Mann et al., 2007). These studies suggest that the skilled players are in a top-down, goal-directed attentional control mode, which can also explain why the skilled perform more efficient anticipation in early occlusion tasks than the unskilled. In contrast, the less-skilled players are likely to rely more on the bottom-up, stimulus-driven attention control mode. Therefore, the skilled are more vulnerable to the adverse effects of high anxiety.

It is worth noting that the effect of high anxiety on attentional control did not differ between the two groups, which is consistent with the findings of Cocks et al. (2016) (based on response accuracy) and inconsistent with the findings of Vater et al. (2016) (based on the eye-movement measure). One possibility is that the skilled and less-skilled players use different attentional strategies (Roca et al., 2013). Particularly, the advantage of the less-skilled being less susceptible to high anxiety is likely offset by the disadvantage of insufficient perceptual-cognitive skills. In addition, the results of Runswick et al. (2018) suggest that the effect of high anxiety on motor performance was limited to the attentional level. However, high anxiety did not affect the cognitive interpretation level and motor behavior level. Therefore, the degree and extent to which high anxiety affects attentional control and multi-level motor performance remain to be further investigated.

Our results re-emphasize the importance to focus on the adverse effects of cognitive state anxiety on athletic performance. Cognitive state anxiety can influence perceptual-cognitive skills, such as anticipation, by disrupting attentional control system. Athletes should balance self-focus (internal long-term working memory) and task-focus (external kinematic cues) under high anxiety, especially for skilled athletes. They should efficiently detach attention from external kinematic cues with the help of the domain-specific knowledge structures. One way is to frequently undergo some training in high anxious or competitive environment in order for athletes to be able to endure high anxiety and have less anxious thoughts (e.g., Pusenjak et al., 2015; Alder et al., 2016). Another method is to develop executive functions, especially shifting and inhibition function (e.g., Ducrocq et al., 2016).

There are some limitations in the current study that should be acknowledged. Although the results show that high state anxiety destroys athletes’ goal-oriented and top-down attentional control, these only speculate on the attention mechanism of athletes during anticipation. Further research should be conducted to investigate how high anxiety affects the attention mechanism, such as the allocation of attention to internal and external stimuli and the change of attentional priority, to broaden and deepen ACT.

In addition, although state anxiety was successfully increased with a combination of manipulations (competitive environment, evaluation threat, error feedback, and monetary reward), there is still a large gap between anxiety induced in a laboratory and anxiety experienced by athletes in a real game. In the future, VR and other technologies can be used to improve the ecological validity of anxiety-induced procedure. Then, researchers should think how to implant the paradigm to a real match. In addition, with the help of video, athletes may possibly evaluate the level of anxiety in a retrospective paradigm for researchers to examine the relationship between anxiety and athletic performance. Moreover, the inclusion of beginners may broaden our understanding on the effect of anxiety on different stages of motor learning and control.

Despite the group-based experimental pattern, the individual zones of optimal functioning (IZOF) model in a sport-specific framework provides us with an individual-based pattern to examine the relationship between emotional experiences and athletic performance (Ruiz et al., 2017). This kind of person-centered design gets rid of the single perspective of intensity of anxiety and emphasize individual differences, such as personal motivation (Ruiz et al., 2017) and personal sport identity (Masten et al., 2006), which would inspire more evidence-based practice that may be especially beneficial for elite athletes.

## Conclusion

In conclusion, using a temporal occlusion paradigm to test ACT in a dynamic anticipation process, we find that high cognitive state anxiety disrupts the goal-directed, top-down attentional control of athletes and reduces their processing efficiency and anticipation performance. The results have implications for the adaptation of attentional strategies in sports competition and daily anxiolytic training.

## Data Availability Statement

The original contributions presented in the study are included in the article/Supplementary Material, further inquiries can be directed to the corresponding author/s.

## Ethics Statement

The studies involving human participants were reviewed and approved by the Ethics Committee of Beijing Sport University. The patients/participants provided their written informed consent to participate in this study. The individual(s) provided their written informed consent for the publication of any identifiable images or data presented in this article.

## Author Contributions

PR: conceptualization, methodology, software, formal analysis, and writing—original draft. TS: investigation, methodology, software, resources, and writing—original draft. LC: supervision, project administration, funding acquisition, and writing—review and editing. XW: visualization and writing—review and editing. XM: data curation and writing—review and editing. All authors contributed to the article and approved the submitted version.

## Conflict of Interest

The authors declare that the research was conducted in the absence of any commercial or financial relationships that could be construed as a potential conflict of interest.

## Publisher’s Note

All claims expressed in this article are solely those of the authors and do not necessarily represent those of their affiliated organizations, or those of the publisher, the editors and the reviewers. Any product that may be evaluated in this article, or claim that may be made by its manufacturer, is not guaranteed or endorsed by the publisher.

## Supplementary Material

Supplementary Table 1 The prime data of the anticipation performance and the scale scores.
